# Mapping the *Shh* long-range regulatory domain

**DOI:** 10.1242/dev.108480

**Published:** 2014-10

**Authors:** Eve Anderson, Paul S. Devenney, Robert E. Hill, Laura A. Lettice

**Affiliations:** MRC Human Genetics Unit, MRC Institute of Genetics and Molecular Medicine, University of Edinburgh, Crewe Rd, Edinburgh EH4 2XU, UK

**Keywords:** Sonic hedgehog (*Shh*), Enhancers, Long-range regulation, Topological domains, Sleeping beauty transposon, Mouse

## Abstract

Coordinated gene expression controlled by long-distance enhancers is orchestrated by DNA regulatory sequences involving transcription factors and layers of control mechanisms. The *Shh* gene and well-established regulators are an example of genomic composition in which enhancers reside in a large desert extending into neighbouring genes to control the spatiotemporal pattern of expression. Exploiting the local hopping activity of the Sleeping Beauty transposon, the *lacZ* reporter gene was dispersed throughout the *Shh* region to systematically map the genomic features responsible for expression activity. We found that enhancer activities are retained inside a genomic region that corresponds to the topological associated domain (TAD) defined by Hi-C. This domain of approximately 900 kb is in an open conformation over its length and is generally susceptible to all *Shh* enhancers. Similar to the distal enhancers, an enhancer residing within the *Shh* second intron activates the reporter gene located at distances of hundreds of kilobases away, suggesting that both proximal and distal enhancers have the capacity to survey the *Shh* topological domain to recognise potential promoters. The widely expressed *Rnf32* gene lying within the *Shh* domain evades enhancer activities by a process that may be common among other housekeeping genes that reside in large regulatory domains. Finally, the boundaries of the *Shh* TAD do not represent the absolute expression limits of enhancer activity, as expression activity is lost stepwise at a number of genomic positions at the verges of these domains.

## INTRODUCTION

The regulatory architecture of highly regulated genes such as those involved in controlling developmental processes has been particularly difficult to define. Identification of regulatory elements by sequence alone has proven difficult; most progress has been made using multispecies conservation and functional analysis such as transgenic mice and DNase sensitivity. In general the regulatory composition of a single gene may consist of multiple elements that reside within introns of the gene and extend to large distances at either end occupying positions in gene deserts and even neighbouring genes. This composition orchestrates layers of control mechanisms, posing a number of questions about the capacity of the regulatory components within these complex regulatory domains.

**Fig. 1.**
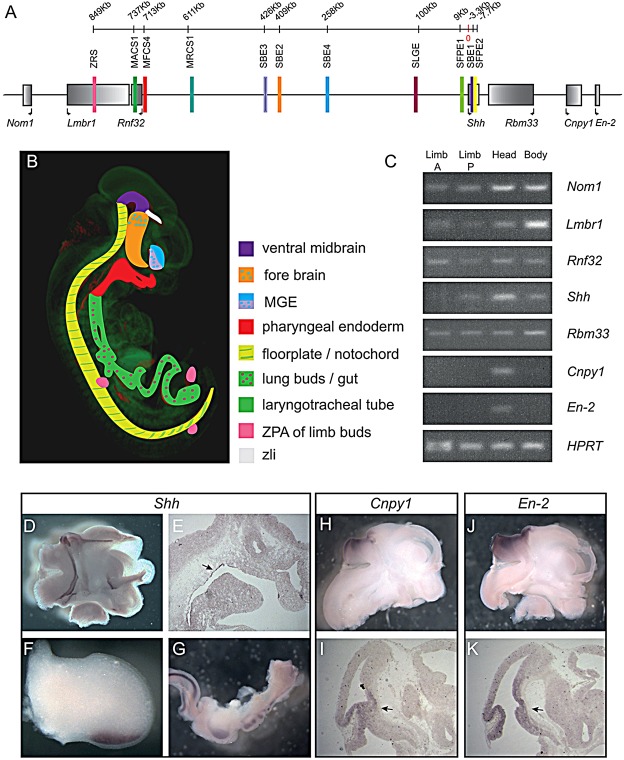
**Expression of genes within the *Shh* regulatory locus.** (A) The genes within the interval from *Nom1* to *En2* are marked by the grey rectangles, shaded from dark to light in the 5′ to 3′ orientation. Known enhancers are shown as coloured bars. (B) Schematic illustration of the multiple sites of *Shh* expression in the E11.5 embryo; the colours used match the relevant enhancers shown in their genomic context in A. Expression in the ZLI is driven by an unknown enhancer. (C) Results of a study of the embryonic expression at E11.5 of those genes conducted by RT-PCR [in anterior and posterior halves of limb buds, in carcass (body) and isolated heads]. *In situ* hybridisation, in E11.5 embryos are shown for *Shh* in whole mount in a bisected head (D), limb (F) and isolated gut (G), and in tissue sections showing expression in the epithelial lining oral cavity (E). Expression of *Cpny1* and *En2* are shown in whole-mount bisected heads (H,J) and in sections (I,K). Expression within the midbrain-hindbrain boundary is marked by arrows.

Sonic hedgehog (*Shh*) is an example of a developmental gene dependent on long-range gene regulatory mechanisms for its full spatiotemporal pattern of expression. With the coding region lying adjacent to a large gene desert, the expression is controlled by a group of *cis*-regulators, many of which were identified by mouse transgenic reporter assays. Epstein et al. (1999) identified two intronic enhancers and one outside the gene, lying ∼9 kb upstream of *Shh*. These potential enhancers of *Shh* activate *lacZ* reporter expression within the ventral midline of the spinal cord and hindbrain and the ventral midbrain and caudal region of the diencephalon. A further study, using comparative sequence analysis and mouse reporter assays, uncovered three forebrain enhancers located 300-450 kb upstream of *Shh* (Jeong et al., 2006). Comparative genomics was also used to identify another more distal cluster of three *cis*-regulatory elements ranging approximately 600-900 kb upstream of *Shh*, one of which lies within an intron of the *Rnf32* gene. This cluster of regulatory elements directs regional expression of *Shh* in a co-linear pattern along the anteroposterior body axis within the epithelial linings of the oral cavity to the hindgut (Sagai et al., 2009). *Shh* initiation and spatial expression control within the posterior margin of the limb bud called the zone of polarizing activity (ZPA) is also regulated by a *cis*-regulatory element designated the ZRS (Lettice et al., 2002, 2003; Sagai et al., 2004, 2009). The ZRS is another intragenic regulator and is found within *Lmbr1*, a gene that lies within a cluster of genes flanking the desert. ZRS is the farthest known enhancer for *Shh*, acting over a distance of nearly 900 kb. Overall the *Shh* genomic regulatory domain comprises nearly 900 kb, containing a number a regulators extending into two unrelated genes, the *Lmbr1* and *Rnf32* genes. (The entire locus and *Shh* expression pattern are summarised diagrammatically in Fig. 1A,B.)

Disruption of the long-range *cis*-regulation of *Shh* causes human congenital defects. Chromosomal rearrangements that disrupt *cis*-regulators act as autosomal dominant mutations to cause a highly variable holoprosecencephaly phenotype (Belloni et al., 1996), whereas chromosomal rearrangements that cause duplications of the ZRS are associated with triphalangeal thumb-polysyndactyly syndrome and syndactyly type IV (Klopocki et al., 2008; Sun et al., 2008). Additionally, point mutations in the ZRS result in ectopic anterior expression of *Shh*, which is a major cause of preaxial polydactyly type 2 (PPD2) (Lettice and Hill, 2005). Furthermore, a large-scale intrachromosomal rearrangement that places *Shh* in a novel regulatory environment, called ‘enhancer adoption’, has been demonstrated to result in a severe limb phenotype in humans (Lettice et al., 2011). A similar gain of regulatory information may explain the brachydactyly phenotype observed in the mouse *Dsh* (short digit) mutant (Niedermaier et al., 2005).

In order to understand coordinated regulatory events responsible for *Shh* expression in the embryo, we modified the endogenous locus. By exploiting the enhancer monitoring aspect of the Local Hopping Enhancer Detection System (LHED) (Kokubu et al., 2009) based on the Sleeping Beauty (SB) transposon, we specifically engineered the chromosomal region containing the *Shh* gene. In this study, we targeted the transposon vector containing the *lacZ* reporter gene into the locus then utilised its transposition capacity to insert extensively throughout the region. This approach enabled us to map enhancer activity throughout the *Shh* regulatory domain to determine a potential relationship between enhancer activity and its position within the locus and elucidate on a chromosomal scale the regulatory events manifested over a long range.

## RESULTS

### Surveillance of the *Shh* regulatory domain

In order to examine the activity of the *Shh* regulatory complex and the long-range activity of *cis*-acting enhancers, we chose to insert reporter genes interspersed throughout the *Shh* regulatory domain on chromosome 5. Vectors were designed based on the LHED strategy (Kokubu et al., 2009), which carries the SB inverted repeats (IRs)/direct repeats (DRs) and combines standard knock-in technology, and transposon based enhancer detection. As our initial concern was to investigate the activity of the most distal enhancer, the ZRS, we inserted the local hopping transposon on either side of this *cis*-regulator. Embryonic stem (ES) cells were generated that contained a targeted integration of the LHED vector within either of the two positions. One, called 5′ Insert, lies 5′ of the ZRS (orientation based on position relative to the *Shh* coding region) about 860 kb from *Shh* and the second, called 3′ Insert, was located on the 3′ side of the ZRS, 781 kb from *Shh*.

Transposon ‘hopping’ was induced in the ES cell lines by transfection of the pCMV-SB100x encoded transposase (Mates et al., 2009). Mobilisation of the transposon confers puromycin resistance, and approximately 200 puromycin-resistant colonies originating from the 5′ Insert ES cells were picked, and 400 colonies from the 3′ Insert ES cells. Reinsertion of the transposon occurred in 60% of 5′-Insert-derived colonies and in 50% of the 3′-Insert-derived colonies. Of the 325 clones where the insertion site was mapped, 50% of the insertion sites were found within chromosome 5, and of these more than 40% were within 1 Mb of the site of origin (summarised in supplementary material Table S2). In addition, the transposon insertion showed no bias in orientation in relation to the site of origin. Sequence of the insertion site from 50 ES cell clones (including those discussed in the text) were analysed for indels generated during the transposition event (data not shown) but no significant insertions or deletions were identified; thus, reinsertion of the transposon appears to be a highly accurate event.

As heterozygous embryos carrying pLHED insertions were sufficient to analyse *cis*-regulatory reporter function, a number of ES cell clones carrying a single insert were selected and injected into tetraploid blastocysts, which generate embryos derived wholly from the ES cells. At E11.5, these embryos were analysed for enhancer activity by examining *lacZ* expression from the transposed transgene.

### Relative expression activity within the gene desert

**Fig. 2.**
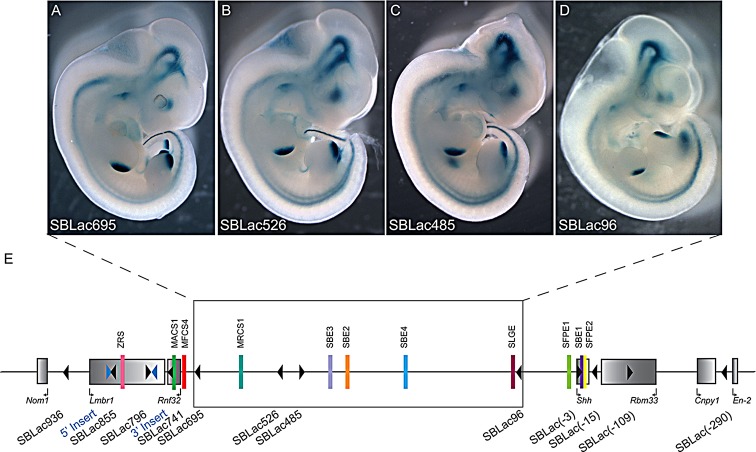
**Expression of SBLac insertions within the gene desert.** (A-D) Embryos derived from the ES cells containing the SBLac re-insertions within the intergenic desert depicted in E, which were harvested at E11.5 and stained for expression of β-gal. The staining observed reflects the *Shh* expression pattern. The diagram in E depicts the genomic interval from *Nom1* to *En2*. In addition to the genes (grey rectangles) and known enhancers (coloured bars), the sites of the original pHLED insertions are marked by the blue triangles and the positions of the mobilised and re-inserted SBLac insertions are marked with black arrowheads. The direction in which the arrowhead points depicts the 5′-to-3′ orientation of the reporter gene. Those SBLac insertions shown in A-D have been boxed.

Initially, an ES cell clone was chosen if the SB transposon insertion site was situated within the gene desert, between the *Shh* and *Rnf32* genes. The gene desert covers ∼730 kb of DNA containing a number of well-characterised *Shh* enhancers responsible for expression in the brain, the floor plate of the neural tube and the epithelial lining of the pharynx and oral cavity. To systematically analyse regulatory activity across this large intergenic region, four ES cell clones were selected that carried reporter genes inserted within the gene desert. Each insert is called SBLac (using the convention of Ruf et al., 2011) and is distinguished by their distance from the transcriptional start of the *Shh* gene; for example, the SBLac96 (*n*=9) lies 96 kb from *Shh* and lies between SFPE1 and SBE4. The other three inserts within the desert that were analysed were SBLac485 (*n*=6), SBLac526 (*n*=7) and SBLac695 (*n*=7), the last sits within the cluster of gut epithelial enhancers between MRCS1 and MFCS4 (Fig. 2E). These clones enabled us to sample enhancer activity across the gene desert as a measure of both the state of the chromatin and the susceptibility of enhancers to genomic distances.

Tetraploid complementation embryos at E11.5 were analysed, and all four reporter insertions showed similar, complex expression patterns (Fig. 2A-D), which compared well to the major sites of *Shh* expression (revealed by *in situ* hybridisation, Fig. 1D-G and previously reported by Echelard et al., 1993; Riddle et al., 1993; Epstein et al., 1999; Chuong et al., 2000). There were no apparent omissions in the patterns of expression. Expression analyses of the genes within the *Shh* interval from *Nom1* to *En2* have been examined by RT-PCR (Fig. 1C), and *in situ* hybridisation in whole mounts and on sections. Specific expression was detected for *Shh* within the brain, pharyngeal endoderm, limb and gut, and for *Cnpy1* and *En2* within the midbrain-hindbrain junction. However, the nearest neighbours to *Shh* (*Nom1*, *Lmbr1*, *Rnf32* and *Rbm33*) all appear to be expressed widely in the embryo (Fig. 1C and data not shown). This widespread expression is supported by embryonic day 14.5 (E14.5) RNA-seq data from the mouse ENCODE project (see webpage, http://genome.ucsc.edu/ENCODE/dataSummaryMouse.html) (The ENCODE Consortium Project, 2011).

**Fig. 3.**
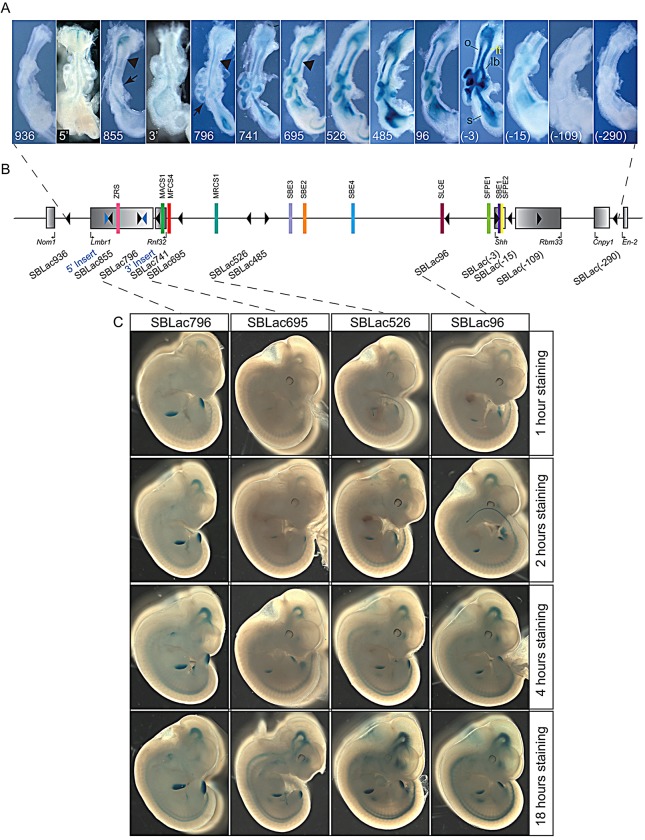
**Expression within guts and a timecourse for β-gal staining.** (A) A series of guts, dissected at E11.5, from embryos derived from cells carrying the initial insertions called 5′ Insert (5′) and 3′ Insert (3′) and the mobilised SBLac containing ES cells throughout the entire interval and stained for β-gal expression. Insertions in the middle of the interval, from SBLac741 to SBLac(-15), reflect the *Shh* expression pattern, with expression being detected in the laryngotracheal tube [in panel labelled (-3)], oesophagus, the lung buds and stomach. The embryonic gut from either end of the interval, SBLac936, SBLac(-109) and SBLac(-290), show no detectable staining. Expression in the laryngotracheal tube, driven by the MACS1 enhancer, is not detected in SBLac855 and SBLac796 (arrowheads). MACS1 shows a directional preference within the regulatory domain, because SBLac695, which lies a similar distance 3′ of MACS1 as SBLac796 lies 5′, shows more activity (expression marked by arrowhead). SBLac855 also shows no expression in the developing lung buds (arrow). (B) The genomic interval, as in Fig. 2E. (C) The timecourse for β-gal staining in embryos from the SBLac796, SBLac695, SBLac526 and SBLac96 insertions, dissected at E11.5 and stained for 1, 2, 4 and 18 h. lb, lung buds; lt, laryngotracheal tube; o, oesophagus; s, stomach.

The analysis for *lacZ* reporter gene activity was done for each embryo under similar β-galactosidase (β-gal) staining conditions such that expression levels were comparable among the different insertion sites. All embryos were stained for 18 h to ensure all sites of expression are detected by the β-gal enzyme reaction. (The consistency of the β-gal staining for similarly staged embryos for two different insertion sites is shown in supplementary material Fig. S1.) To ensure that the final staining pattern reflected the levels of expression, we followed the timecourse of staining for four different insertions within the Shh regulatory domain; SBLac 796, SBLac695, SBLac526 and SBLac96 (Fig. 3C). The timecourse included embryos at 1, 2, 4 and 18 h and showed that qualitative comparison of expression levels is possible between the insertion sites. The progression of staining in the early time points reflects the relative pattern at the 18 h time end point.

Analysis of the expression patterns for the gene desert inserts suggest that the desert is in an open conformation over its length and each reporter is receptive to all the known enhancer activities. In addition, the insert position did not affect the pattern of expression and none of the reporters showed unusual or unexpected additions to the expression pattern as would be expected for a holo-enhancer (Marinic et al., 2013).

### Distance as a modulator of enhancer activity

Although the pattern of expression was not affected by position of the reporter within the regulatory domain, the levels of expression showed positional differences. The question of how location of an SB reporter insert pertains to expression levels was addressed by focusing on the activity of two of the enhancers, ZRS and MACS1, which are at the extreme 5′ end of the regulatory domain. These two enhancers afford the opportunity to examine enhancer activity over a large genomic distance.

The MACS1 enhancer is responsible for widespread expression in the epithelium of the gut, stomach, alveoli and laryngotracheal tube, and lies inside the *Rnf32* gene (Fig. 1A). A nonconserved secondary enhancer, called SLGE, lies within 100 kb 5′ of the *Shh* gene and drives expression that overlaps the MACS1 pattern in all tissues except the laryngotracheal tube [labelled in yellow in Fig. 3A, panel marked (-3)] which has no known secondary enhancer (Tsukiji et al., 2014). Analysis of the four reporters between MACS1 and the *Shh* gene lying within the gene desert (Fig. 3) showed levels of expression within the laryngotracheal tube, which appeared to be highest in SBLac485. Surprisingly, expression of the two nearest reporter genes, SBLac695 and SBLac741, which lie within the *Rnf32* gene and at 5 kb away are the closest insertions to MACS1, showed that proximity to the MACS1 enhancer has no influence on expression activity. The MACS1 enhancer also has an apparent directional preference within the regulatory domain; the two reporter insertions on the 5′ side (SBLac796 and SBLac855) show very low activity in the laryngotracheal tube. SBLac796, which is 60 kb on the 5′ side of MACS 1, is a similar distance to the more active SBLac695 (∼40 kb), which lies on the opposite side (compare the expression marked by the black arrowheads in SBLac796 and SBLac695 in Fig. 3). Additionally, no activity is detectable in the SBLac855 (arrowhead), suggesting a gradual loss of MACS1 activity at this side of the enhancer.

**Fig. 4.**
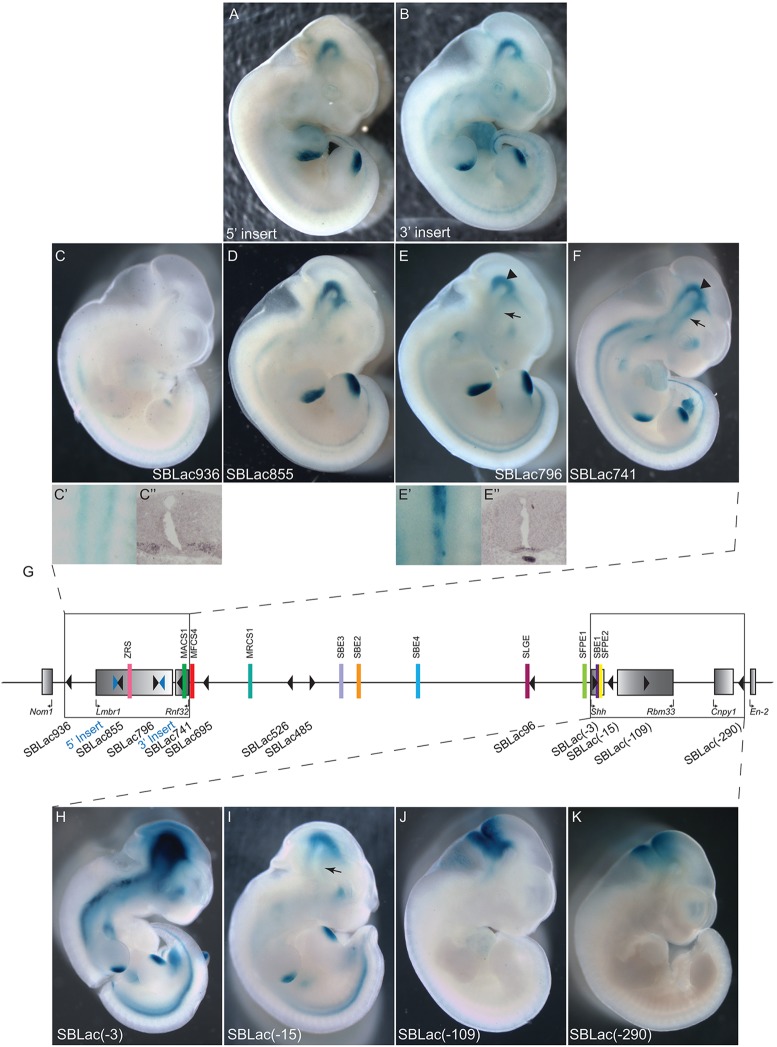
**Expression of SBLac insertions at the extreme ends of the *Shh* regulatory locus.** (A,B) Embryos derived from ES cells carrying the initial insertion sites and (C-F) from SBLac-carrying ES cells from the 5′ end of the regulatory domain. SBLac936 shows faint *Shh* like expression in the limb buds but the staining down the back is detected in two stripes (C′), which does not reflect the *Shh* pattern in the floor plate and notochord (E′), but more likely reflects expression of *Mnx1*, the next gene past *Nom1*. Expression of *Mnx1* is shown by *in situ* hybridisation (C″). *In situ* hybridisation for *Shh* expression in the floor plate and notochord is shown in E″. Expression in SBLac855, SBLac796 and SBLac741 does reflect the *Shh* pattern; however, expression is lost preferentially from the developing forebrain in SBLac855 and SBLac796, whereas expression in the midbrain is maintained. [Compare expression marked by arrows (forebrain), with that marked by arrowheads (midbrain) in E,F.] (G) The regulatory locus with the relevant SBLac insertions (black triangles) boxed. At the 3′ end of the region (H-K), highest levels of expression are detected in SBLac(-3), which has integrated within the coding region of *Shh*. Expression in the next insertion, SBLac(-15), is maintained in most of the sites of *Shh* expression but at much lower levels, and expression is completely missing from the forebrain (arrow). However, in the last two insertions, SBLac(-109) and SBLac(-290), expression is detected only at the midbrain-hindbrain junction, mirroring expression of *Cnpy1* and *En2*, and suggesting these SBLac insertions have integrated within a different topological domain.

The ZRS is the enhancer that maps the furthest distance from the *Shh* gene, lying in intron 5 of the *Lmbr1* gene. The spatiotemporal information that drives the limb expression is contained in this single enhancer; a previous report shows that deletion of this region results in loss of limb expression (Sagai et al., 2005). The two insertions, SBLac796 (*n*=12) and SBLac855 (*n*=15), sit to either side of the ZRS and are the nearest reporter genes to this enhancer. In contrast to the low expression of these two SBLac insertions in the gut, and laryngotracheal tube (Fig. 3A), the expression in the limb is appreciable, confirming that this region of the domain is open for enhancer activity (Fig. 4D-F). Other insertions between the ZRS and *Shh* also showed expression, but differences were difficult to discern, as the limb buds stain appreciably at the 18 h time point. To compare expression across the regulatory domain, expression in the limb buds was analysed over the timecourse in Fig. 3C. The limb bud expression in SBLac796 was compared to the three insertions within the gene desert, SBLac695, SBlac526 and SBLac96. SBLac796 staining is readily seen in the limb buds at 1 h and appears to approach saturation by 4 h of staining, whereas the insertions within the gene desert lying at a greater distance lag behind. SBlac695, SBLac526 and SBlac96 show increasing limb staining (see Fig. 3) the closer the reporter lies to the *Shh* gene. In contrast to the MACS1 enhancer, the ZRS exhibits appreciably higher activity with reporters that are located nearby. This activity falls dramatically in the proximal gene desert and then increases gradually again with proximity to *Shh*.

### Expression susceptibility inside an ubiquitously expressed gene

The *Rnf32* gene is expressed widely throughout the embryo, but no developmental role has, thus far, been assigned. The *Rnf32* gene has *Shh* enhancers upstream, (the ZRS limb-specific enhancer resides in intron 5 of the *Lmbr1* gene) downstream and residing inside the gene itself (the MACS1 enhancer, discussed above, resides inside intron 8). However, the *Rnf32* gene expression is not detectably upregulated in the gut or any other regions that reflect the *Shh* pattern (Fig. 1C and data not shown). To determine whether there is a property integral to the *Rnf32* gene that renders it unresponsive to the local regulatory landscape, we selected an insertion, SBLac741, which is located in intron 6 of the Rnf32 gene. SBLac741 is expressed throughout the central nervous system (CNS), brain, gut and limb in a typical *Shh* spatial pattern (Fig. 4F). Therefore *Rnf32*, which resides inside the regulatory domain and carries an active promoter (data from ENCODE summarised in Fig. 6B), is resistant to outside enhancer influence; however, the body of the gene is accessible to the surrounding expression activity. Although the *Shh* enhancers are not limited to the *Shh* promoter, as evidenced by the activity of the heterologous promoter of the reporter genes, it appears that the Rnf32 promoter is refractory to these activities.

### Activity of regulators that reside within the *Shh* gene

Two enhancers reside within the introns of the *Shh* gene. These enhancers work at the shortest distance and, by operating within the context of the gene, avoid the need for any participation with the upstream regulatory domain. This gave us the opportunity to assay the intragenic enhancer activity to determine whether intronic enhancers have similar characteristics to long-range-acting enhancers. The floor plate enhancer SFPE2 in exon 3 overlaps the activity of a similar enhancer in the gene desert at E11.5, the SFPE1, and therefore activity was difficult to specifically attribute to SFPE2 (Fig. 1B). We therefore focused on the SBE1 enhancer, which is located in intron 2 of the *Shh* gene and is responsible for *Shh* expression in the ventral midline of the rostral midbrain and caudal diencephalon (Fig. 1B). Mice carrying a 525 bp targeted deletion of the SBE1 enhancer initiate *Shh* expression at E8.5 in these brain regions but are unable to maintain it after E10.0 (Jeong et al., 2011). By contrast, *Shh* expression within the forebrain tissue, the zona limitans intrathalamica (ZLI), is unaffected. These data suggest that SBE1 is required for expression in the ventral midbrain at E11.5 and thus presents a distinctive expression pattern that can be used to analyse enhancer function. We found that all SBLac reporters in the gene desert (Fig. 2A-D) and residing in the *Rnf32* and *Lmbr1* genes (Fig. 4A,B,D-F) are under the influence of the ventral midbrain SBE1 regulator, as reporter gene expression is present within the midbrain (although not the ZLI in some instances), showing that this enhancer, although near the *Shh* promoter, has the information to act at a very long distance of over 700 kb. Therefore, enhancer proximity to the promoter and residence inside the gene does not indicate a different class of regulator and underscores the idea that mechanisms are shared by intragenic enhancers and long-distance enhancers.

### Defining the boundaries of *Shh* enhancer activity

We observed that reporter gene activity within *Lmbr1* decreased differentially in a subset of *Shh*-expressed tissues. For example, between SBLac741 and SBLac796 there is little change in production in the ventral midbrain (driven by the SBE1 enhancer) (indicated by arrowheads in Fig. 4E,F), whereas the forebrain expression (SBE2 enhancer) is noticeably reduced (indicated by arrows in Fig. 4E,F). Also the laryngotracheal expression (MACS1 enhancer) is drastically reduced between the same two insertion sites (arrowheads in Fig. 3A). The lung expression dependent on the MACS1 and SLGE enhancers is dramatically reduced between SBLac796 and SBLac855 (arrows in Fig. 3A).

To examine the possibility that orientation of the insertion, especially those located inside active genes, may affect the relative expression of reporters, we compared five different insertion sites within genes at the *Lmbr1* end of the regulatory domain. SBLac741in the *Rnf32* gene and in the *Lmbr1* gene, SBLac796 and SBLac855, and the insertion sites of the initial transposon constructs, 5′ Insert and 3′ Insert were examined. SBLac 855 and the 5′ Insert and SBLac796 and the 3′ Insert are nearest neighbours and are situated in opposite orientations (Fig. 3B). Relative expression appears to be dependent on position and not on orientation (compare gut expression in Fig. 3A and expression in whole embryos in Fig. 4A,D and Fig. 4B,E). For example, the laryngotracheal tract and forebrain expression is present in SBLac741 but not in the inserts in the *Lmbr1* gene, regardless of orientation.

In order to determine how far *Shh* enhancer activities persist within the chromosomal domain, embryos were generated containing an SBLac insertion located 24 kb upstream of the *Lmbr1* gene (SBLac936). The SBLac936 insertion was situated between *Lmbr1* and the neighbouring *Nom1* gene. *In situ* hybridisation and RT-PCR (Fig. 1C and data not shown) showed that *Nom1* was expressed widely throughout the embryo with no discernible *Shh* pattern. SBLac936 (*n*=5) expression was undetectable throughout most of the embryo, except within the neural tube and the limb. The limb expression mirrors that of *Shh* in the ZPA and is probably due to residual activity from the ZRS (Fig. 4C). However, dissected embryos showed that the neural tube expression was unlikely to be *Shh* related given that expression was found in two lateral stripes corresponding to the motoneurons (Fig. 4C′), rather than within the *Shh* midline domain in the floor plate and notochord (Fig. 4E′). This probably reflects expression of *Mnx1*, which is the next gene past *Nom1*. (For comparison, expression of *Mnx1* by *in situ* hybridisation is shown in Fig. 4C″, whereas *Shh in situ* is shown in Fig. 4E″.) No other expression resembling the *Shh* pattern was found.

**Fig. 5.**
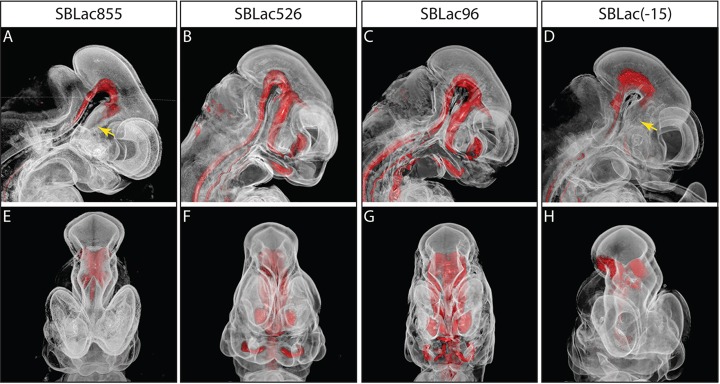
**OPT analysis of SBLac insertions.** Outputs of OPT analysis of E11.5 embryos derived from SBLac insertions across the regulatory locus [SBLac855 (A,E), SBLac526 (B,F), SBLac96 (C,G) and SBLac(-15) (D,H)]. Sagittal views are shown in A-D and frontal views in E-H. The anatomy of the samples is translucent, whereas the β-gal expression is coloured red. Expression is confirmed to be missing from the forebrains of SBLac8555 and SBLac(-15) (yellow arrows), whereas midbrain expression is maintained.

SBLac insertions were also examined downstream of *Shh* (Fig. 4G): firstly, SBLac[-15] (*n*=10) within the intergenic region between *Shh* and the nearest neighbouring gene *Rbm33*, approximately 15 kb from the *Shh* 5′ end; and secondly, an insertion within an intronic region of the *Rbm33* gene SBLac[-109] (*n*=7). Finally, the furthest 3′ insertion studied, SBLac[-290] (*n*=6), lies between *Cnpy1* and *En2* (Fig. 4H-K). The expression pattern of SBLac[-15] was shown to reflect that of the *Shh* pattern within the CNS, gut and limbs; however, levels were appreciably lower than the adjacent insertion, SBLac[-3]. SBLac[-3] (*n*=5) shows the highest levels of expression observed, but this insertion resides within the *Shh* gene and lies in the orientation of transcription. In addition, the forebrain expression driven by the SBE2 enhancer was undetectable (arrow in Fig. 4I). We used optical projection tomography (OPT) (Sharpe et al., 1999) to examine this expression in SBLac[-15] compared to SBLac96 and SBLac526 to gain a 3D view of the expression (Fig. 5B-D,F-H), which agreed with the relative loss of expression in the forebrain. The lack of forebrain expression observed in SBLac[-15] (Fig. 5D,H) is also very similar to that at the opposite 5′ end of the regulator domain in SBLac855 (Fig. 5A,E), suggesting that a similar mechanism might be at play at both extreme ends. Focusing on the ventral midbrain enhancer (SBE1) that resides inside the *Shh* gene, which has no promoter lying between it and the reporter in SBLac[-15], also shows lower activity (Fig. 4I), suggesting that downregulation of activity within the *Shh* regulatory domain is not due to the promoter generating an interfering boundary; however, promoter preference cannot be ruled out. The SBE1 enhancer is capable of working bidirectionally; however, action in the opposite direction from the target gene is severely limited. The more distal insertions SBlac[-109] and SBLac[-290] showed no *Shh* related expression; instead expression was found within the midbrain-hindbrain junction. Thus a distinctive boundary exists between SBLac[-15] and SBLac[-109] but similar to loss of activity at the 5′ end of the regulatory domain, expression is initially reduced before becoming undetectable.

The midbrain/hindbrain expression patterns observed in SBlac[-109] and SBLac[-290] support the notion that these two reporter genes were influenced by the same regulatory element but within a domain distinct from that of *Shh*. Downstream of *Shh* lie three genes: *Rbm33*, *Cnpy1* and *En2*. Very little is known about the first gene other than sequence composition suggesting an RNA-binding activity. *Cnpy1* and *En2*, however, are expressed within the midbrain-hindbrain boundary region (Fig. 1H-K) and *En2* plays a role in restricting the fate of progenitor cells to a midbrain or hindbrain lineage (Davis et al., 1988; Paek et al., 2012). The expression of SBlac[-109] and SBLac[-290] appears to be under the control of the midbrain-hindbrain regulatory element. SBLac[-109] lies inside the *Rbm33* gene, presenting a second example of a gene resistant to outside regulatory influence, similar to the observations made for the *Rnf32* gene. This raises the possibilities that promoter resistance plays a general role within large regulatory domains that encompass multiple genes.

## DISCUSSION

The analysis of the *Shh* regulatory locus took advantage of the previous transgenic and genetic analyses that established the contribution of the highly conserved elements on the spatial expression activities (Epstein et al., 1999; Lettice et al., 2002, 2003; Sagai et al., 2004, 2009; Jeong et al., 2006). Most of these *Shh* regulators operate at mid-gestation; thus, generation of tetraploid complementation embryos focusing on a single stage (∼E11.5) in development proved to be an effective assay for enhancer activity across a large chromosomal domain.

Coordinated gene expression controlled by long-distance enhancers is recognised to fall into a number of different, but possibly overlapping, mechanistic classes. Regulatory landscapes that control the output of the HoxD gene cluster rely on multiple inputs from regulatory elements scattered across hundreds of kilobases (called regulatory archipelagos) that control sequential expression of genes in the HOX cluster as well as the co-expression of the ‘bystander’ genes *Lnp* and *Evx2* (Spitz et al., 2003; Montavon et al., 2011). The holo-enhancer of FGF8 requires multiple regulatory elements, many of which lie inside genes acting together to generate a complex developmental expression pattern. The enhancer activity and the expression pattern are highly dependent on the position of the gene relative to the regulatory elements, suggesting a complex interaction of the regulatory elements. These regulators do not activate neighbouring genes (Marinic et al., 2013). By contrast, *Shh* developmental activity may represent a more common genomic composition (Symmons and Spitz, 2013) relying on a regulatory domain primarily composed of regulators that contribute to the spatiotemporal expression pattern as a summation of the individual enhancer activities. The enhancers that regulate *Shh* expression populate a gene desert but extend past this desert into two genes of a neighbouring gene-rich region. As with holo-enhancers, neighbouring gene expression is unaffected.

### Gene desert is open to enhancer activity

Reporter genes inserted into any of the four positions of the 729 kb gene desert displayed spatial patterns that reflected *Shh* expression upon examination of the whole embryo and the dissected gut. Long-range activity is inbuilt into the enhancers assayed in the *Shh* region. The gene desert is broadly open to transcriptional activity; and the spatial pattern of expression was independent of the position of the reporters within the desert, suggesting that there is no segmentation of the regulatory terrain into open and closed chromatin and that the chromatin within the gene desert is functionally indistinguishable.

Long-range acting enhancers are believed to have adopted mechanisms that enable the interaction of enhancers with their respective promoters by a looping mechanism, which is cytologically visible by 3D fluorescence *in situ* hybridisation (FISH) (Bickmore, 2013). In support of these mechanisms the most distal *Shh* regulator, the ZRS enhancer, was shown to employ these interactions (Amano et al., 2009) and these were related to gene activity (Lettice et al., 2014). If these mechanisms are crucial for long-range activity, the question arises as to how these relate to enhancers acting over much shorter distances. Notably, the ventral midbrain enhancer that resides within the *Shh* gene and operates on the target promoter only 6 kb away has full capacity to recognise reporter genes up to a distance of ∼800 kb. This relationship of enhancer and promoter interactions to gene activity suggest uniformity in enhancer mechanisms across the whole regulatory domain and indicates that proximity does not necessarily require the implementation of different mechanisms. Although this approach does not indicate if there is a promoter preference, it does show that heterologous promoters are accessible by the *Shh* enhancers. These enhancers are fully capable of recognising promoters in numerous positions within the regulatory domain, suggesting that enhancers possess a surveillance activity that bidirectionally scans across broad regions of the regulatory domain.

### Activity level differs dependent on position

Although the majority of the gene desert appears to be open to enhancer activity, the activity levels are not equivalent across the domain. We examined the most distal enhancer, the ZRS limb-specific enhancer lying in the *Lmbr1* gene, which showed a trend in which the reporter closest to the enhancer was high with a decrease in expression toward the middle of the domain and an increase with the reporters near the *Shh* gene. This increase in pHLED-derived reporter activity nearer the gene was also reported for the *Pax1* gene (Kokubu et al., 2009). Alternatively, the MACS1 gut enhancer is found within the *Rnf32* gene (Tsukiji et al., 2014) and expression driven by MACS1 in the laryngotracheal tube suggests a mechanism more focused in the direction of the gene but does not increase with proximity to the *Shh* gene.

### Resistance to enhancers by widely expressed genes

The *Rnf32* gene lies fully within the *Shh* regulatory domain and carries one of the known enhancers within an intron. Expression of *Rnf32*, however, does not reflect the *Shh* expression pattern but is expressed broadly throughout the embryo at E11.5. One possible explanation for this resistance to the *Shh* regulatory domain is that the gene is outside regulatory influence; however, the insertion inside the gene has the full capacity to generate the *Shh* expression pattern. In accordance, this regulatory evasion may be a common mechanism, as a similar resistance to nearby enhancer activity was shown for the *Rbm33* gene. Both the *Rbm33* and *Rnf32* genes appear to be fully within range of enhancer influence, and we suggest that the promoters are refractory to enhancer activity, perhaps highlighting a common event within large regulatory domains.

### Topological domains and the limits of enhancer activity

**Fig. 6.**
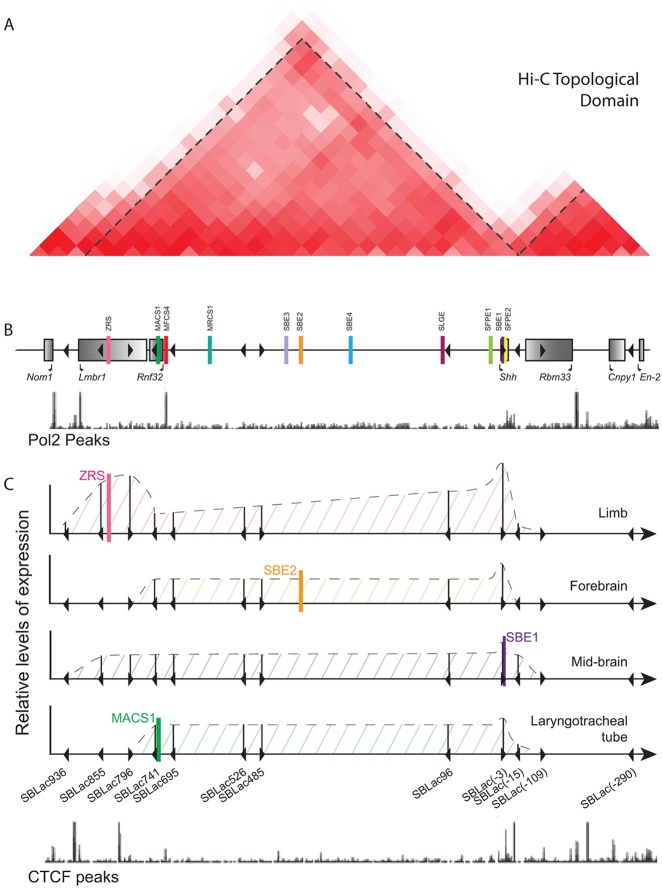
**Summary of the limits of topological domains and enhancer activity.** (A) The Hi-C analysis from mouse ES cells (taken from the Mouse ENCODE website), and the boundaries of the topological domain determined by the directionality index and marked by dotted lines. The relationship between the positions of these features and the genes and insertions within the *Nom1* to *En2* region are indicted in B. Below the genes is the track showing the positions of RNA polymerase II (Pol2 peaks marked with black lines) in E14.5 limb buds taken from ENCODE. (C) A summary of relative expression activity driven by individual enhancers in particular tissues (ZRS driving expression in the limb bud, SBE2 in the forebrain, SBE1 in the midbrain and MACS1 in the laryngotracheal tube) at each of the SBLac insertions. The solid vertical lines show an estimate of the relative expression levels and the dotted lines the predicted levels throughout the genomic interval. Expression at the 5′ end shows that reduction in expression occurs over an interval of greater than 100 kb, whereas limb bud expression is still detectable at the furthest insertion site. Positions of CTCF peaks (bright blue lines) in E14.5 limb buds taken from ENCODE are shown below. There are no appreciable peaks in the middle of the interval, the majority lying at either end; however, there is no relationship between position of the peaks and the position at which expression is reduced.

We have mapped the extent of the regulatory domain responsible for the spatiotemporal expression of the *Shh* gene. The *Shh* enhancers were shown to operate within a single long-range chromosomal domain with boundaries that limit enhancer activity. On a large genomic scale, chromatin interaction studies using Hi-C techniques suggest that the genome is arranged into large megabase-sized ‘topological domains’ or ‘topological associated domains’ (TADS), the boundaries of which are expected to correspond to insulator or barrier elements, which prevent the spread of heterochromatin (Smallwood and Ren, 2013). Furthermore, a genome-wide functional study examining randomly integrated reporters shows that large regulatory domains are confined to TADs and that enhancers generally act pervasively throughout these regulatory domains (Symmons et al., 2014). The *Shh* regulatory domain agrees with the TAD previously predicted (Fig. 6A) (Smallwood and Ren, 2013). However, distinct boundaries that block enhancer activity within these domains are not absolute. At the *Lmbr1* end of the domain enhancer, function is found to tail off in a stepwise fashion within the gene (depicted in Fig. 6C). The laryngotracheal expression is drastically reduced between SBLac741 and SBLac796, whereas the expression in the lung primordial is lost between SBLac796 and SBLac855. Finally, the ventral midbrain and the limb expression levels are drastically reduced between SBLac855 and SBLac936, but low levels of *Shh* in the limb are still detectable. At the *Shh* end of the regulatory domain, enhancer activity is reduced 15 kb downstream of the gene promoter, whereas the forebrain expression is undetectable. All expression is lost at the next insert within the *Rbm33* gene. These data suggest that there are no absolute limits to enhancer function and a number of barriers exist that downregulate expression.

TAD boundary regions are postulated to be enriched for the insulator binding protein CTCF (Dixon et al., 2012) that prevent illegitimate enhancer-promoter interactions**.** The major CTCF sites identified in the analysis of E14.5 limbs (Shen et al., 2012) are shown in Fig. 6C. Within the *Shh* and *Lmbr1* region such sites were identified (ENCODE), and these sites correspond well with the end of the TAD mapped by Hi-C (Smallwood and Ren, 2013). Notably, no CTCF sites lie within the gene desert (Fig. 6C). The incremental loss of expression toward the limits of the *Shh* topological domain tallies with multiple CTCF sites; however, there is no direct relationship between the position of the CTCF sites and the loss of enhancer activity, suggesting that additional mechanisms limit the activity of enhancers within a regulatory domain.

## MATERIALS AND METHODS

### Targeting vector construction

Two vectors were designed for targeted integration into the *Lmbr1* locus: the first a 7.7 kb homology arm at the 5′ end of the *Lmbr1* gene, 7.5 kb upstream of the ZRS; and the second a 7.6 kb arm 74 kb downstream of the ZRS. Mini targeting arms were generated by PCR (the primers are listed in supplementary material Table S1) and cloned using the underlined restriction sites into pBluescript (Invitrogen). Bacterial recombineering (Liu et al., 2003) was then employed to retrieve the homology arms from the Pac RCPI-21 542n10 (Osoegawa et al., 2000). These homology arms were subcloned (using the bold restriction sites) into the pLHED vector (Kokubu et al., 2009). The constructs had been designed with gaps, of 600 and 666 bp, respectively, flanked by *Hin*dIII sites that were used to linearise the vectors for targeting.

### Cell culture and gene targeting

E14TG2a ES cells were cultured and targeted using standard techniques (Lettice et al., 1999). 100 µg of each linearised vector were electroporated into 1×10^7^ ES cells. After 10 days of selection with G418, individual clones were picked, screened for correct targeting by PCR (primers are listed in supplementary material Table S1) and the insertion position confirmed by DNA FISH.

### *In vitro* mobilisation of transposon

LHED targeted cells were plated at densities of 1×10^6^ per 10 cm plate, transiently transfected with 20 µg of the transposase vector pCMV-SB100x (Mates et al., 2009), using TransFast (Promega) and cultured for 48 h before the addition of puromycin (2 µg/ml). After 10 days of selection, resistant colonies were picked into 96-well plates, grown and DNA produced for analysis. Excision of the transposon was confirmed by PCR using the primers for Puromycin gene and the PGK promoter. Reinsertion of transposon was detected using *lacZ*-specific primers.

### Identifying transposon insertion sites

To identify transposon insertion sites, a nested asymmetric PCR strategy was applied as described by Ruf et al. (2011) (primers are listed in supplementary material Table S1).

### Embryo production, whole-mount X-gal staining and *in situ* hybridisation

Tetraploid blastocysts were produced by electrofusion, and ES cells injected to make entirely ES-cell-derived embryos by tetraploid complementation (Nagy et al., 1993). Whole-mount X-gal staining of embryos was performed at E11.5 as described previously (Lettice et al., 2003), but on this occasion the staining was allowed to proceed at room temperature for between 1 and 18 h in a concentration of 300 µg/ml X-gal.

Wild-type mouse embryos were harvested at E11.5 and *in situ* hybridisation was performed with DIG-labelled gene-specific antisense probes as previously described (Hecksher-Sorensen et al., 1998).

Probes were generated for *Lmbr1*, *Rnf32*, *Nom1* and *Rbm33* by RT-PCR and cloned into the pBluescript vector (Agilent Technologies) (primers are listed in supplementary material Table S1). The *Shh* probe was kindly provided by Andy McMahon (Echelard et al., 1993) and the *Cnpy1* and *En2* probes by Jean M Herbert (Paek et al., 2012).

### Gene expression analysis by RT-PCR

RNA was extracted from E11.5 mouse embryos by using RNAbee (AMSBio) and cDNA was made using a First-Strand cDNA Synthesis Kit (Roche). Primers (listed in supplementary material Table S1) were exon specific.

### OPT analysis

OPT imaging was performed (Sharpe et al., 2002). Briefly, PFA-fixed embryos were embedded in 1% low-melting-point agarose and then immersed in methanol for 24 h to remove all water. The embryo was then cleared for 24 h in BABB (one part benzyl alcohol/two parts benzyl benzoate). The sample was then scanned using a Bioptonics 3001 scanner (www.biooptonics.com) with an image taken every 0.9° (of a 360° rotation). Upon completion, images are reconstructed using Bioptonics proprietary software with the outputs then being viewed with Dataviewer (Bioptonics) and Bioptonics Viewer. Additional, 3D outputs were produced using Drishti rendering software (Ajay Limaye-Volume Exploration and Presentation Tool).

## Supplementary Material

Supplementary Material

## References

[DEV108480C1] Amano, T., Sagai, T., Tanabe, H., Mizushina, Y., Nakazawa, H. and Shiroishi, T. (2009). Chromosomal dynamics at the Shh locus: limb bud-specific differential regulation of competence and active transcription. *Dev. Cell* 16, 47-57 10.1016/j.devcel.2008.11.011 19097946

[DEV108480C2] Belloni, E., Muenke, M., Roessler, E., Traverso, G., Siegel-Bartelt, J., Frumkin, A., Mitchell, H. F., Donis-Keller, H., Helms, C., Hing, A. V. et al. (1996). Identification of Sonic hedgehog as a candidate gene responsible for holoprosencephaly. *Nat. Genet.* 14, 353-356 10.1038/ng1196-353 8896571

[DEV108480C3] Bickmore, W. A. (2013). The spatial organization of the human genome. *Annu. Rev. Genomics Hum. Genet.* 14, 67-84 10.1146/annurev-genom-091212-153515 23875797

[DEV108480C4] Chuong, C.-M., Patel, N., Lin, J., Jung, H.-S. and Widelitz, R. B. (2000). Sonic hedgehog signaling pathway in vertebrate epithelial appendage morphogenesis: perspectives in development and evolution. *Cell. Mol. Life Sci.* 57, 1672-1681 10.1007/PL00000650 11130174 PMC4381998

[DEV108480C5] Davis, C. A., Noble-Topham, S. E., Rossant, J. and Joyner, A. L. (1988). Expression of the homeo box-containing gene En-2 delineates a specific region of the developing mouse brain. *Genes Dev.* 2, 361-371 10.1101/gad.2.3.361 2454212

[DEV108480C6] Dixon, J. R., Selvaraj, S., Yue, F., Kim, A., Li, Y., Shen, Y., Hu, M., Liu, J. S. and Ren, B. (2012). Topological domains in mammalian genomes identified by analysis of chromatin interactions. *Nature* 485, 376-380 10.1038/nature11082 22495300 PMC3356448

[DEV108480C7] Echelard, Y., Epstein, D. J., St-Jacques, B., Shen, L., Mohler, J., McMahon, J. A. and McMahon, A. P. (1993). Sonic hedgehog, a member of a family of putative signaling molecules, is implicated in the regulation of CNS polarity. *Cell* 75, 1417-1430 10.1016/0092-8674(93)90627-3 7916661

[DEV108480C8] Epstein, D. J., McMahon, A. P. and Joyner, A. L. (1999). Regionalization of Sonic hedgehog transcription along the anteroposterior axis of the mouse central nervous system is regulated by Hnf3-dependent and -independent mechanisms. *Development* 126, 281-292. 9847242 10.1242/dev.126.2.281

[DEV108480C9] Hecksher-Sorensen, J., Hill, R. E. and Lettice, L. (1998). Double labeling for whole-mount in situ hybridization in mouse. *Biotechniques* 24, 914–916, 918. 9631179 10.2144/98246bm02

[DEV108480C10] Jeong, Y., El-Jaick, K., Roessler, E., Muenke, M. and Epstein, D. J. (2006). A functional screen for sonic hedgehog regulatory elements across a 1 Mb interval identifies long-range ventral forebrain enhancers. *Development* 133, 761-772 10.1242/dev.02239 16407397

[DEV108480C11] Jeong, Y., Dolson, D. K., Waclaw, R. R., Matise, M. P., Sussel, L., Campbell, K., Kaestner, K. H. and Epstein, D. J. (2011). Spatial and temporal requirements for sonic hedgehog in the regulation of thalamic interneuron identity. *Development* 138, 531-541 10.1242/dev.058917 21205797 PMC3014638

[DEV108480C12] Klopocki, E., Ott, C.-E., Benatar, N., Ullmann, R., Mundlos, S. and Lehmann, K. (2008). A microduplication of the long range SHH limb regulator (ZRS) is associated with triphalangeal thumb-polysyndactyly syndrome. *J. Med. Genet.* 45, 370-375 10.1136/jmg.2007.055699 18178630

[DEV108480C13] Kokubu, C., Horie, K., Abe, K., Ikeda, R., Mizuno, S., Uno, Y., Ogiwara, S., Ohtsuka, M., Isotani, A., Okabe, M. et al. (2009). A transposon-based chromosomal engineering method to survey a large cis-regulatory landscape in mice. *Nat. Genet.* 41, 946-952 10.1038/ng.397 19633672

[DEV108480C14a] Lettice, L. A. and Hill, R. E. (2005). Preaxial polydactyly: a model for defective long-range regulation in congenital abnormalities. *Curr. Opin. Genet. Dev.* 15, 294-300 10.1016/j.gde.2005.04.002 15917205

[DEV108480C14] Lettice, L. A., Purdie, L. A., Carlson, G. J., Kilanowski, F., Dorin, J. and Hill, R. E. (1999). The mouse bagpipe gene controls development of axial skeleton, skull, and spleen. *Proc. Natl. Acad. Sci. USA* 96, 9695-9700 10.1073/pnas.96.17.9695 10449756 PMC22272

[DEV108480C15] Lettice, L. A., Horikoshi, T., Heaney, S. J. H., van Baren, M. J., van der Linde, H. C., Breedveld, G. J., Joosse, M., Akarsu, N., Oostra, B. A., Endo, N. et al. (2002). Disruption of a long-range cis-acting regulator for Shh causes preaxial polydactyly. *Proc. Natl. Acad. Sci. USA* 99, 7548-7553 10.1073/pnas.112212199 12032320 PMC124279

[DEV108480C16] Lettice, L. A., Heaney, S. J. H., Purdie, L. A., Li, L., de Beer, P., Oostra, B. A., Goode, D., Elgar, G., Hill, R. E. and de Graaff, E. (2003). A long-range Shh enhancer regulates expression in the developing limb and fin and is associated with preaxial polydactyly. *Hum. Mol. Genet.* 12, 1725-1735 10.1093/hmg/ddg180 12837695

[DEV108480C17] Lettice, L. A., Daniels, S., Sweeney, E., Venkataraman, S., Devenney, P. S., Gautier, P., Morrison, H., Fantes, J., Hill, R. E. and FitzPatrick, D. R. (2011). Enhancer-adoption as a mechanism of human developmental disease. *Hum. Mutat.* 32, 1492-1499 10.1002/humu.21615 21948517

[DEV108480C18] Lettice, L. A., Williamson, I., Devenney, P. S., Kilanowski, F., Dorin, J. and Hill, R. E. (2014). Development of five digits is controlled by a bipartite long-range cis-regulator. *Development* 141, 1715-1725 10.1242/dev.095430 24715461 PMC3978833

[DEV108480C19] Liu, P., Jenkins, N. A. and Copeland, N. G. (2003). A highly efficient recombineering-based method for generating conditional knockout mutations. *Genome Res.* 13, 476-484 10.1101/gr.749203 12618378 PMC430283

[DEV108480C20] Marinić, M., Aktas, T., Ruf, S. and Spitz, F. (2013). An integrated holo-enhancer unit defines tissue and gene specificity of the Fgf8 regulatory landscape. *Dev. Cell* 24, 530-542 10.1016/j.devcel.2013.01.025 23453598

[DEV108480C21] Mates, L., Chuah, M. K. L., Belay, E., Jerchow, B., Manoj, N., Acosta-Sanchez, A., Grzela, D. P., Schmitt, A., Becker, K., Matrai, J. et al. (2009). Molecular evolution of a novel hyperactive Sleeping Beauty transposase enables robust stable gene transfer in vertebrates. *Nat. Genet.* 41, 753-761 10.1038/ng.343 19412179

[DEV108480C22] Montavon, T., Soshnikova, N., Mascrez, B., Joye, E., Thevenet, L., Splinter, E., de Laat, W., Spitz, F. and Duboule, D. (2011). A regulatory archipelago controls Hox genes transcription in digits. *Cell* 147, 1132-1145 10.1016/j.cell.2011.10.023 22118467

[DEV108480C23] Nagy, A., Rossant, J., Nagy, R., Abramow-Newerly, W. and Roder, J. C. (1993). Derivation of completely cell culture-derived mice from early-passage embryonic stem cells. *Proc. Natl. Acad. Sci. USA* 90, 8424-8428 10.1073/pnas.90.18.8424 8378314 PMC47369

[DEV108480C24] Niedermaier, M., Schwabe, G. C., Fees, S., Helmrich, A., Brieske, N., Seemann, P., Hecht, J., Seitz, V., Stricker, S., Leschik, G. et al. (2005). An inversion involving the mouse Shh locus results in brachydactyly through dysregulation of Shh expression. *J. Clin. Invest.* 115, 900-909 10.1172/JCI200523675 15841179 PMC1070420

[DEV108480C25] Osoegawa, K., Tateno, M., Woon, P. Y., Frengen, E., Mammoser, A. G., Catanese, J. J., Hayashizaki, Y. and de Jong, P. J. (2000). Bacterial artificial chromosome libraries for mouse sequencing and functional analysis. *Genome Res.* 10, 116-128. 10645956 PMC310499

[DEV108480C26] Paek, H., Antoine, M. W., Diaz, F. and Hébert, J. M. (2012). Increased beta-catenin activity in the anterior neural plate induces ectopic mid-hindbrain characteristics. *Dev. Dyn.* 241, 242-246 10.1002/dvdy.22787 22102609 PMC3266450

[DEV108480C27] Riddle, R. D., Johnson, R. L., Laufer, E. and Tabin, C. (1993). Sonic hedgehog mediates the polarizing activity of the ZPA. *Cell* 75, 1401-1416 10.1016/0092-8674(93)90626-2 8269518

[DEV108480C28] Ruf, S., Symmons, O., Uslu, V. V., Dolle, D., Hot, C., Ettwiller, L. and Spitz, F. (2011). Large-scale analysis of the regulatory architecture of the mouse genome with a transposon-associated sensor. *Nat. Genet.* 43, 379-386 10.1038/ng.790 21423180

[DEV108480C29] Sagai, T., Masuya, H., Tamura, M., Shimizu, K., Yada, Y., Wakana, S., Gondo, Y., Noda, T. and Shiroishi, T. (2004). Phylogenetic conservation of a limb-specific, cis-acting regulator of Sonic hedgehog (Shh). *Mamm. Genome* 15, 23-34 10.1007/s00335-033-2317-5 14727139

[DEV108480C30] Sagai, T., Hosoya, M., Mizushina, Y., Tamura, M. and Shiroishi, T. (2005). Elimination of a long-range cis-regulatory module causes complete loss of limb-specific Shh expression and truncation of the mouse limb. *Development* 132, 797-803 10.1242/dev.01613 15677727

[DEV108480C31] Sagai, T., Amano, T., Tamura, M., Mizushina, Y., Sumiyama, K. and Shiroishi, T. (2009). A cluster of three long-range enhancers directs regional Shh expression in the epithelial linings. *Development* 136, 1665-1674 10.1242/dev.032714 19369396

[DEV108480C32] Sharpe, J., Lettice, L., Hecksher-Sørensen, J., Fox, M., Hill, R. and Krumlauf, R. (1999). Identification of sonic hedgehog as a candidate gene responsible for the polydactylous mouse mutant Sasquatch. *Curr. Biol.* 9, 97-100 10.1016/S0960-9822(99)80022-0 10021368

[DEV108480C33] Sharpe, J., Ahlgren, U., Perry, P., Hill, B., Ross, A., Hecksher-Sørensen, J., Baldock, R. and Davidson, D. (2002). Optical projection tomography as a tool for 3D microscopy and gene expression studies. *Science* 296, 541-545 10.1126/science.1068206 11964482

[DEV108480C34] Shen, Y., Yue, F., McCleary, D. F., Ye, Z., Edsall, L., Kuan, S., Wagner, U., Dixon, J., Lee, L., Lobanenkov, V. V. et al. (2012). A map of the cis-regulatory sequences in the mouse genome. *Nature* 488, 116-120 10.1038/nature11243 22763441 PMC4041622

[DEV108480C35] Smallwood, A. and Ren, B. (2013). Genome organization and long-range regulation of gene expression by enhancers. *Curr. Opin. Cell Biol.* 25, 387-394 10.1016/j.ceb.2013.02.005 23465541 PMC4180870

[DEV108480C36] Spitz, F., Gonzalez, F. and Duboule, D. (2003). A global control region defines a chromosomal regulatory landscape containing the HoxD cluster. *Cell* 113, 405-417 10.1016/S0092-8674(03)00310-6 12732147

[DEV108480C37] Sun, M., Ma, F., Zeng, X., Liu, Q., Zhao, X.-L., Wu, F.-X., Wu, G.-P., Zhang, Z.-F., Gu, B., Zhao, Y.-F. et al. (2008). Triphalangeal thumb-polysyndactyly syndrome and syndactyly type IV are caused by genomic duplications involving the long range, limb-specific SHH enhancer. *J. Med. Genet.* 45, 589-595 10.1136/jmg.2008.057646 18417549

[DEV108480C38] Symmons, O. and Spitz, F. (2013). From remote enhancers to gene regulation: charting the genome's regulatory landscapes. *Philos. Trans. R. Soc. Lond. B Biol. Sci.* 368, 20120358 10.1098/rstb.2012.0358 23650632 PMC3682723

[DEV108480C39] Symmons, O., Uslu, V. V., Tsujimura, T., Ruf, S., Nassari, S., Schwarzer, W., Ettwiller, L. and Spitz, F. (2014). Functional and topological characteristics of mammalian regulatory domain. *Genome Res.* 24, 390-400 10.1101/gr.163519.113 24398455 PMC3941104

[DEV108480C40] The ENCODE Consortium Project. (2011). A user's guide to the encyclopedia of DNA elements (ENCODE). *PLoS Biol.* 9, e1001046 10.1371/journal.pbio.1001046 21526222 PMC3079585

[DEV108480C41] Tsukiji, N., Amano, T. and Shiroishi, T. (2014). A novel regulatory element for Shh expression in the lung and gut of mouse embryos. *Mech. Dev.* 131, 127-136 10.1016/j.mod.2013.09.003 24157522

