# Supplementary material for: Mapping the Shh long-range regulatory domain
Source: Development. 2014 Oct;141(20):3934–43. doi: 10.1242/dev.108480 (PMC4197689; doi:10.1242/dev.108480)
Supplement: Supplementary Material [file supp_141_20_3934__index.html]

Mapping the Shh long-range regulatory domain — Supplementary Material 

# Mapping the *Shh* long-range regulatory domain

## DEV108480 Supplementary Material

**Files in this Data Supplement:**

- **Supplementary Material**
