# Supplementary material for: Mapping the Shh long-range regulatory domain
Source: Development. 2014 Oct;141(20):3934–43. doi: 10.1242/dev.108480 (PMC4197689; doi:10.1242/dev.108480)
Supplement: Supplementary Material [file supp_141.20.3934_DEV108480.pdf]

Figure S1- shows 2 examples of a number tetraploid complementation embryos which were derived from the same ES line. This demonstrates the consistancy of the staining pattern and its intensity between embryos.

Anderson et al., Supplementary Figure 1

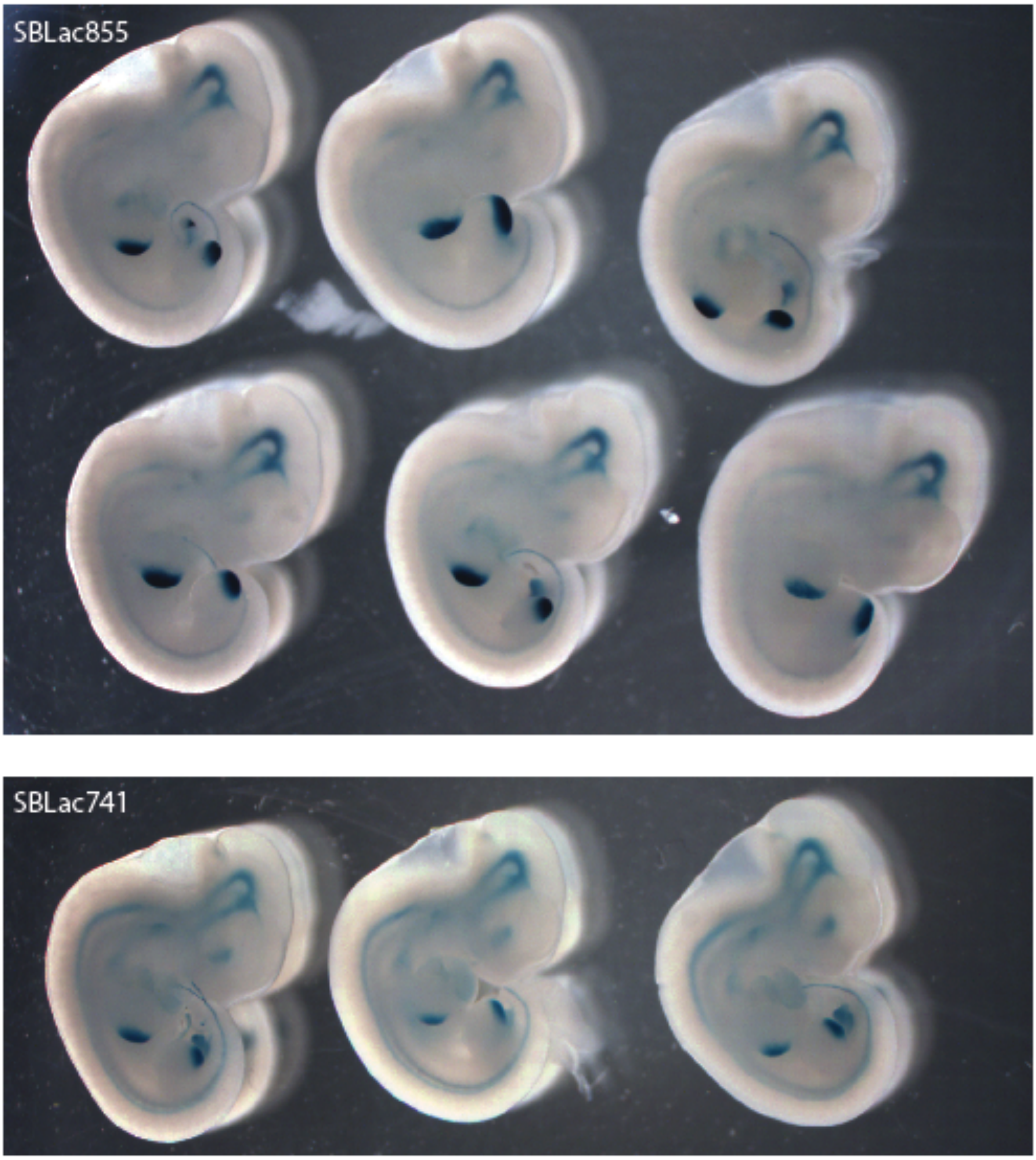

Table S1- PCR primers

Targeting Vector Primers		
Vector 1	arm 1(F)<br><br>(R)	GATCATGCGGCCGC**GGCGCGCC**AAGCAAAGCACCAGCCATT<br><br>GATCATATCGAT GACCAATGAGCTCCAAGGAT
	arm 2(F)<br><br>(R)	GATCATATCGATCCAAGCCTCAGCTGTTCTTC<br><br>GATCATCTGCAG**TTAATTAA**AGGCAGTGAAGGATTTGGAA
Vector 2	arm 1(F)<br><br>(R)	GATCATGCGGCCGC**TTAATTAA**CCACATCCGATAGAGGGCTA<br><br>GATCATATCGATAAGCTTGTTTGCAATCCTACCAGCAA
	arm 2(F)<br><br>(R)	GATCATATCGATAAGCTTGGGCCTCATCAAGGAATTTT<br><br>GATCATGTCGAC**GGCGCGCC**CTCATGAGCCCTTCCATACTG
Screening of ES cell clones for correct targeting		
	M13	TGTAAAACGACGGCCAGTGAGC
	Neo	GCCTTCTATCGCCTTCTTCTTGACG
	3'F2	GCCATGTTAGTCACATCATGCAC
	3'R2	CATTTACCACATCCCTAGCTGTG
	5'F2	TCAAGTCTGACTAGGATAACTTAG
	5'R2	TAGTTTGAGCCTTAGGTGTGCAC
Screening of the SB transposon re-insertion		
	Puro:<br>PGK	CGTGGGCTTGTACTCGGTC<br>GACAGCACCGCTGAGCAATG
	Lac(F)<br>(R)	CAACTTAATCGCCTTGCAGCAC<br>CGCTGATTTGTGTAGTCGGTT
Identifying the pHLED re-insertion site		
	SBR1	CTTCTGACCCACTGGGAATGTGATG
	SBR2	GTGGTGATCCTAACTGACCTAAGAC
	SBR3	TCCTAACTGACCTAAGACAGG
	SBL1	CTGGAATTGTGATACAGTGAATTATAAGTG
	SBL2	CTTGTGTCATGCACAAAGTAGATGTCC
	SBL3	AAGTAGATGTCCTAACTGACTTGC
	KmonP-N7-CTCAG	GTACGAGAATCGCTGTCCTNNNNNNNCTCAG
	KmonP-N7-TCCTG	GTACGAGAATCGCTGTCCTNNNNNNNTCCTG
	KmonP	GTACGAGAATCGCTGTCCT
Cloning the in situ probes		
	Nom1	GATCATGTCGACGGCTCAGGTTCTGAGACTCG<br>GATCATGCGGCCGCGTCAACACCCTCCGTAGGAA
	Rnf32	GATCATGTCGACTCAGCCATGCCCAATATGTA<br>GATCATGCGGCCGCACAGATGTGCACAGGACAGG,
	Rbm33	GATCATGTCGACGGGCAACACTTGAGACCATT

		GATCATGCGGCCGCCTGGACATCAGTGGTGGATG,
	Lmbr1	GATCATGTCGACCTGTGATGTCCAGAACACTG<br>GATCATGCGGCCGCGATTCTGTAAGATGAATCAG.
RT-PCR analysis		
	Nom1	GGCTCAGGTTCTGAGACTCG<br>GTCAACACCCTCCGTAGGAA
	Lmbr1	GCTGGTTGATGAGACTGCAA<br>GTGCTTTCTGATGCCCATTT
	Rnf32	TCAGCCATGCCCAATATGTA<br>ACAGATGTGCACAGGACAGG
	Shh	GCCTACAAGCAGTTTATTCCCAAC<br>CAGTGGATGTGAGCTTTGGATTC
	Rbm33	GGGCAACACTTGAGACCATT<br>CTGGACATCAGTGGTGGATG
	Cnpy1	GCGAATGAACGATTACCAG<br>AATAAGTTCGAATATCTCATCTTC
	En2	CTGCACGCGCTATTCTGAC<br>GAGACTCGTTCAGGCTGAG
	HPRT	CACAGGACTAGAACACCTGC<br>GCTGGTGAAAAGGACCTCT

Table S2 – Position of the SBLac insertions obtained within 1Mb of the initial insertion.

Insertion	Chromosome	strand	Start site
7	5	neg	28787130
13	5	neg	29445046
46	5	pos	29116092
SBLac(-290)	5	neg	28505362
68	5	neg	29639709
92	5	pos	29444355
98	5	pos	28797914
102	5	pos	29630364
104	5	neg	29622149
109	5	pos	28994110
115	5	pos	28978743
118	5	neg	29634908
SBLac96	5	neg	28889280
130	5	neg	29583183
SBLac485	5	pos	29279078
148	5	pos	29066794
148	5	pos	29066794
156	5	neg	29870513
189	5	pos	29116275
198	5	pos	29647904
SBLac855	5	neg	29648912
SBLac741	5	neg	29535017
232	5	pos	29797578
SBLac526	5	neg	29319708
277	5	pos	29266136
288	5	pos	29011233
302	5	neg	28996239
305	5	pos	29498715
SBLac936	5	neg	29729576
SBLac(-109)	5	pos	28684534
330	5	neg	29798033
SBLac(-15)	5	neg	28777971
360	5	pos	29794971
SBLac(-3)	5	pos	28790598
376	5	pos	29403698
392	5	neg	29653752
393	5	neg	28996029
393	5	neg	28996029
396	5	pos	29003367
397	5	neg	29654566

2112	5	pos	29471537
SBLac695	5	neg	29489073
2134	5	pos	29515622
2157	5	neg	29591086
2161	5	pos	29472020
2179	5	pos	29475308
2191	5	neg	29468681
SBLac796	5	pos	29589741
3109	5	neg	29649217
3119	5	pos	29810217
3137	5	pos	29626757
3139	5	neg	29653746
